# An AI-Assisted Review of Trends in Environmental and Human Health

**DOI:** 10.1055/s-0046-1816602

**Published:** 2026-11-03

**Authors:** George S. Hanna, Dmitry A. Scherbakov, Jeffrey R. Guyon, Alexander Bakumenko, Alexander V. Alekseyendo

**Affiliations:** 1 Medical University of South Carolina, Department of Public Health Sciences, Hollings Marine LaboratoryCharleston SC 29412USA; 2 Medical University of South Carolina, Biomedical Informatics CenterCharlestonSC29403USA; 3 National Oceanic and Atmospheric Administration, National Ocean Service, National Centers for Coastal Ocean Science, Hollings Marine LaboratoryCharlestonSC29412USA; 4 Clemson University, School of ComputingClemsonSC29634USA

**Keywords:** Machine learning, Citizen science, Drug discovery, Bioinformatics, Marine health

## Abstract

**Objectives:**

This narrative review uses generative artificial intelligence (AI) tools based on large language models (LLMs) to summarize scientific literature related to environmental and human health. It evaluates three distinct methodologies for knowledge synthesis using LLMs and investigates applications of informatics in citizen science, environmental health, and drug discovery.

**Methods:**

Three approaches were tested. The first approach was a reading list-based review using preselected citations and Google NotebookLM to assess machine learning and citizen science applications to environmental health. The second approach was an AI-assisted narrative review utilizing scite.ai to synthesize data on drug discovery from natural environmental sources. The third approach was a conversational LLM-based synthesis employing ChatGPT to explore bioinformatics in marine and medical sciences, generating new research questions and thematic areas.

**Results:**

AI tools helped summarize the literature and conclude that machine learning combined with citizen science improves environmental data collection and analysis through the use of low-cost sensors and public data annotation efforts. On the drug discovery end, natural products remain a vital source, with advancements in genome mining and cheminformatics enhancing research efficiency. Finally, marine bioinformatics links environmental and human health, identifying shared challenges like pollution impacts and microbial resilience.

**Conclusions:**

Generative AI tools demonstrate potential in scientific literature synthesis as evidenced by their ability to identify relevant application of data science and informatics in environment and human health applications. Some challenges, such as some omitted references and the need to iterate through the prompts necessitate the need for expert supervision of these tools.

## 1. Introduction

The anthroposphere, or the environment surrounding humans, is both a source of threats and cures to ailments. In this narrative review, we aim to identify knowledge at the intersection of informatics and data science with applications in environmental health. By exploring three available Large Language Model (LLM)-based writing tools and evaluating the results, this perspective review was designed to assist readers in selecting the most appropriate tool to accomplish literature reviews to inform hypothesis ideation, concept significance determination and rapid literature summarization.

LLM tools for performing literature reviews are becoming widely used but differ in their capacity to accomplish specific tasks [ 1 ]. LLMs signify a notable advancement in the field of review automation, following earlier methodologies rooted in machine learning (ML) and natural language processing (NLP) that laid the groundwork for assisted reviewing and annotation [ 2 ]. Prior methods to automate reviews primarily focused on text classification and data extraction, aiming to decrease the manual review burden and improve review efficiency [ 3 , 4 ]. While these efforts achieved significant efficiencies in screening and extraction stages, their adoption was constrained by concerns about bias, limited annotated datasets, and accuracy in highly specific tasks, making their usage difficult in new domains and reviews. LLMs have since qualitatively expanded these capabilities.

Our overall goals behind this review are:

(i) Demonstrate approaches for knowledge extraction using LLM tools: Google NotebookLM (Google, Mountain View, CA), scite.ai (Research Solutions, Brooklyn, NY), and ChatGPT (OpenAI, San Francisco, CA);
(ii) Summarize key aspects of current literature on three topics of interest:
  - Machine learning and citizen science in environmental health;
  - Drug discovery from the environment;
  - Applications of bioinformatics in marine and medical sciences.

## 2. Methods and Results

A graphical summary of the three AI/LLM methodologies is provided in Figure 1 .

**Figure 1.**
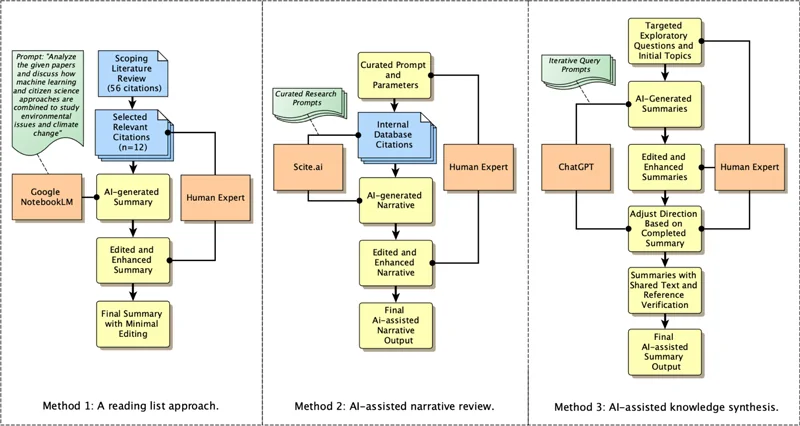
The usage of AI/LLM during review creation for the three methods/reviews.

All of the applied methods resulted in either a complete compact review (Methods 1 and Methods 2), or some preparatory text or section ideas for the prospective review (Method 3). We present the results by using Italics font in the output to display AI-generated content, and regular font to denote edits, inserts, and changes done by the authors.

### 2.1 Citizen Science, Machine Learning, and the Environment: a Reading List Approach

This review creation method uses citations identified during a previously published scoping review on machine learning and citizen approaches in healthcare [ 5 ]. The completed review identified 52 citations, covering different health areas, from cardiology to epidemiology. The present review aims to repurpose the 12 citations corresponding to environmental health to answer the question of how machine learning and citizen science approaches are used to study environmental issues. To conduct this review, we used Google NotebookLM. After uploading 12 papers in full-text PDF format we issued the following prompt:

“Analyze the given papers and discuss how machine learning and citizen science approaches are combined to study environmental issues and climate change.”

The resulting text was evaluated by a human expert (DS), minimally formatted, and is presented below.

*Citizen science and machine learning are increasingly being combined to study environmental issues and climate change, offering novel approaches to data collection and analysis [ 6 – 9 ].*

#### Data Collection and Generation.

Citizen science engages the public in scientific investigations, allowing for data collection over larger areas and time scales, and generating large databases of information [ 8 ]. For example, the FreshWaterWatch project uses citizen scientists to collect water quality data [ 8 ]. In another study, citizens used low-cost sensors to gather air pollution data [ 10 ]. Additionally, the “Sons al Balcó” project engaged citizens to record environmental sounds [ 11 ]. These citizen science projects often utilize low-cost sensors and mobile technologies [ 6 , 7 , 10 , 12 ], such as smartphone-based particle counters [ 12 ], custom-made ozone sensing devices [ 7 ], and air quality monitoring devices [ 10 ]. This facilitates data collection at a finer spatial resolution than traditional monitoring methods [ 12 ]. The data collected by citizens is often combined with remote sensing data or other publicly available data [ 8 ]. For example, in one study, ground-level nitrogen dioxide (NO2) measurements from a citizen science project were combined with aerial images [ 9 ]. Another study combined citizen science water quality data with online GIS [Geographic Information System] information [ 8 ].

#### Data Analysis and Modeling.

*Machine learning techniques, such as deep learning and random forests, are used to analyze the data collected by citizen scientists [ 8 – 10 , 12 ]. These methods can identify patterns and relationships in the data, which may not be apparent through traditional statistical methods [ 7 , 8 ]. Deep learning models, particularly convolutional neural networks (CNNs), are used to analyze images and audio data collected in citizen science projects [ 9 , 11 , 13 ]. For instance, CNNs are used to estimate outdoor NO2 concentrations from aerial images [ 9 ], predict perceptions of the built environment from street-view images [ 13 ], and classify sound events from environmental audio recordings [ 11 ]. Random forest models are used for various tasks such as predicting water quality based on citizen science data [ 8 ], estimating air pollution concentrations [ 12 ], and assessing the impact of lockdown measures on NO2 levels [ 14 ]. Machine learning methods are also used to fuse data from multiple sources and to improve the accuracy of predictions. For example, the multiview data fusion model (MVDF) combines spatial and temporal views of air pollution data [ 10 ]. Machine learning is also used to calibrate low-cost sensors with standard pollution measuring devices [ 7 , 10 ].*

**Table:** 

The following three prompts were used:
What are the major environmental sources for natural product drug discovery? What are important new data sources, new technologies, and new analytical methods for natural product drug discovery from environmental sources and what are potential problems with them? What are understudied areas, ongoing challenges and future frontiers of environmental natural product drug discovery research?

#### Applications and Benefits.

*Air Quality Monitoring: These combined approaches have been used to estimate NO2 concentrations [ 6 , 9 , 14 ], map urban air quality [ 10 , 12 , 15 ], predict particulate matter (PM2.5) concentrations [ 9 , 10 , 12 , 15 ], and monitor ozone levels [ 7 ].*

*Water Quality Assessment: Citizen science and machine learning are used to assess water quality in urban streams and study the drivers of water quality degradation [ 8 ].*

*Environmental Perception: Machine learning models have been trained using crowd-sourced image comparisons to quantify perceptions of the built environment, such as nature quality, beauty, relaxation, and safety [ 13 ].*

*Noise Pollution: Citizen science and machine learning approaches have also been applied to analyzing soundscapes, classifying acoustic events [ 11 ] and exploring the spatiotemporal variability of noise pollution [ 10 ].*

*Impact Assessment: These methods are also used to evaluate the impact of policy interventions, such as the effect of COVID-19 lockdowns on air quality [ 6 , 14 ] and the health impacts of air pollution [ 6 , 14 ].*

*Public Awareness and Engagement: Citizen science projects increase public awareness of environmental issues and enable citizens to participate in solving local problems [ 6 , 8 , 10 ]. The use of low-cost sensors and readily available data contributes to this effort [ 7 , 8 ].*

*In conclusion, the combination of citizen science and machine learning is proving to be a powerful approach for studying environmental issues and climate change. Citizen science provides large amounts of data, while machine learning provides the tools to analyze these complex datasets effectively.*

#### Expert summary.

Citizen science and machine learning approaches have their own applications but can be combined to achieve remarkable results in terms of democratization of research. Machine learning is becoming increasingly accessible with a growth of online learning platforms such as DataCamp and Coursera, while low-cost sensors and the spread of the Internet of Things (IoT) devices allow the general public to participate more in citizen science environmental projects.

### 2.2. Data and Technologies for Drug Discovery from the Environment: AI-assisted Narrative Review

For this section, the smart citation indexing tool chosen was scite.ai , which uses traditional document ingestion and machine learning to analyze over 25 million full-text scientific articles and has a database of over 880 million citation statements [ 16 ]. The default settings scite.ai were used (create review using 25 references, medium length response output). Three prompts were developed and two inferences using each prompt were run in scite.ai to compile a structured review that (i) covers a broad background on the field of natural product drug discovery; (ii) describes advances in analytical, informatics, and technological approaches; and (iii) as a conclusion, explores knowledge gaps, challenges and new directions that can be leveraged for future research.

#### [Prompt 1, Iteration 1]

*Natural product drug discovery has its roots deeply embedded in the exploration of diverse environmental sources, including terrestrial, marine, and microbial ecosystems. These sources are pivotal due to their rich biodiversity, which has historically yielded numerous therapeutic agents. A significant proportion of modern pharmaceuticals, approximately 34% of medicines approved by the FDA between 1981 and 2010, are natural products or their derivatives, underscoring the importance of environmental sources [ 17 – 19 ]. Terrestrial plants have been a primary focus, the complex secondary metabolites produced by plants have been instrumental in developing various therapeutic agents. The integration of ethnobotanical knowledge, which involves studying plants used by indigenous cultures, has also proven beneficial in identifying potential drug candidates [ 20 ]. For instance, the analgesic properties of aspirin can be traced back to compounds found in the Salix genus, which was used medicinally by Native Americans [ 21 ]. Beyond this well-known example, the historical use of plants in traditional medicine has provided a foundation for many modern drugs that have been validated through contemporary scientific methods [ 22 , 23 ].*

*Independent from ethnobotanical knowledge, marine environments represent another reservoir of natural products. Marine organisms, including macroalgae, sponges, other invertebrates and microorganisms, produce unique metabolites that have shown promise in drug development. Due to their diverse chemical properties, marine natural products have led to the approval of several drugs and have revealed novel biological mechanisms [ 24 , 25 ]. Microbial sources, particularly bacteria and fungi, are critical in the search for new drugs. The discovery of antibiotics such as penicillin from fungi, Penicillium chrysogenum, illustrates a significant contribution of microbial natural products to modern medicine [ 26 , 27 ]. Despite the historical success of microbial-derived drugs, there remains a vast untapped potential, with estimates suggesting that 90% of microbial biodiversity has yet to be investigated for drug discovery [ 28 ]. A central effort to provide researchers access to the vast diversity of natural products is the National Cancer Institute’s initiative to create a publicly accessible library of natural product fractions. This program aims to enhance the chemical diversity available for high-throughput screening, emphasizing the need to explore marine biodiversity further [ 28 ]. The development of advanced screening techniques and computational methods has facilitated the identification and isolation of bioactive compounds from these sources, making it easier to transition from screening hits to viable drug candidates [ 29 , 30 ].*

#### [Prompt 2 Iteration 1]

*The evolution of natural product research increasingly relies on new data sources, technologies, and analytical methods. Continued advancements promise to enhance the identification and development of novel therapeutic agents, although they also present several challenges. One of the most significant is the application of genome mining techniques. This approach leverages the sequencing of microbial genomes to uncover previously hidden natural products by analyzing gene clusters associated with secondary metabolite biosynthesis. As noted by Zhang, the potential of microbial natural products is vastly underestimated, and genome mining can significantly enhance the discovery process by identifying novel compounds that could serve as drug candidates [ 31 ]. Additionally, the integration of cheminformatics has become crucial, as it facilitates the curation and analysis of vast chemical databases, allowing researchers to predict the bioactivity and safety profiles of natural products through in silico methods [ 32 ]. Advancements in mass spectrometry and other analytical techniques have revolutionized the structural elucidation of complex natural products. These innovations enable researchers to analyze complex mixtures at a single-cell resolution, which is essential for understanding the bioactive compounds present in environmental samples [ 33 ]. The application of analytical methods such as gas chromatography-mass spectrometry (GC-MS), nuclear magnetic resonance (NMR), and high-performance liquid chromatography (HPLC) remains fundamental in the characterization of natural products [ 34 ]. These methods allow for the detailed analysis of chemical structures and the assessment of their pharmacological activities, which is critical for drug development. Furthermore, the emergence of 3D shape-based approaches enhances the ability to predict macromolecular targets for complex natural products, thus improving the efficiency of the drug discovery pipeline [ 32 ].*

*The use of AI and machine learning in drug discovery is another transformative trend, providing powerful tools for predicting biological effects and optimizing lead compounds [ 35 ]. These technologies can streamline the drug discovery process, reducing the time and resources required to identify promising candidates.*

#### [Prompt 3, Iteration 1]

*Looking toward the future, the exploration of microbial sources, particularly those from extreme environments such as deep-sea ecosystems and arid regions continues to present new opportunities for discovery because the exploration of these environments remains limited [ 19 , 27 ]. The chemical diversity found in lesser-known plant species, particularly species endemic to biodiverse regions, is often overlooked. The Dictionary of Natural Products has cataloged an array of plant secondary metabolites, yet many of these compounds have not been thoroughly investigated for their pharmacological potential [ 27 ]. The potential of natural products derived from marine organisms is still largely untapped. Marine biodiversity harbors unique chemical entities that could serve as novel drug leads, particularly for diseases that are currently difficult to treat [ 22 ]. The integration of advanced screening technologies and computational methods could significantly enhance the discovery of these underexplored natural products [ 36 ].*

*Despite the promising potential of natural products in drug discovery, several challenges persist. One major issue is the technical difficulty associated with the isolation and characterization of bioactive compounds from complex natural extracts. Traditional methods often result in low yields and can be time-consuming [ 30 ]. The dereplication of known compounds remains a significant hurdle, as many natural product extracts contain a plethora of structurally similar compounds, complicating the identification of novel entities [ 28 ]. Another challenge is the declining interest and investment in natural product research within the pharmaceutical industry. Many companies have shifted focus towards synthetic compounds, leading to a reduction in in-house natural product research capabilities [ 37 ]. This trend poses a risk of losing valuable insights and potential drug leads that could be derived from natural sources. Additionally, ethical and economic barriers related to bioprospecting and the sustainable use of biodiversity present ongoing challenges. The Convention on Biological Diversity emphasizes the need for equitable sharing of benefits arising from the utilization of genetic resources, which can complicate research initiatives [ 38 , 39 ].*

#### [Prompt 2, Iteration 2]

*The complexity of natural products often leads to difficulties in their characterization and the reproducibility of results, which can hinder the progression from discovery to clinical application [ 40 ]. While AI and cheminformatics offer promising solutions, they also come with limitations, such as the need for high-quality and centralized data and the risk of generating false positives in lead identification [ 36 ]. The reliance on computational models necessitates a careful balance between in silico predictions and experimental validation to ensure the reliability of findings [ 32 , 41 – 43 ].*

#### [Prompt 3, Iteration 2]

*Looking ahead, the future of environmental natural product drug discovery is promising, particularly with the advent of innovative technologies. The application of computational biology, including molecular modeling and virtual screening, is expected to streamline the identification of bioactive compounds and enhance the efficiency of drug discovery campaigns [ 44 ]. The integration of high-throughput screening methods, such as native mass spectrometry, allows for the rapid assessment of thousands of natural products, facilitating the identification of drug-like candidates [ 30 ]. The resurgence of interest in ethnopharmacology and traditional medicine is likely to drive the exploration of natural products derived from indigenous knowledge systems. This approach not only enriches the chemical diversity available for drug discovery but also promotes the conservation of biodiversity [ 45 , 46 ]. Collaborative efforts between academia, industry, and local communities can foster sustainable practices in bioprospecting and ensure the ethical use of natural resources [ 38 , 39 ]. By addressing the existing gaps in research, leveraging modern technologies, and fostering ethical practices, the field can continue to thrive and contribute to the development of new drugs.*

#### Expert summary

Natural product research continues to advance by exploring the vast diversity of ocean ecosystems and microbial influences on biochemistry, with implications for human health. Combining ethnobotanical knowledge with modern screening technologies and computational biology enhances drug discovery efficiency, enabling the translation of traditional medicines into clinical applications.

### 2.3. Omics Applications in Marine Research: AI-assisted Knowledge Synthesis

To investigate a conversational LLM interface, ChatGPT-4o and GPT-4.5 (reference OpenAI. ChatGPT, 20 Mar. 2023, https://chat.openai.com/chat ) were utilized to summarize bioinformatics research linking marine environments to human health. This interactive approach facilitated an evolving discussion that generated research priorities through two primary conversations (Appendix 1 and Appendix 2). Throughout the process, human intervention was required to refine and expand upon ChatGPT’s responses, simulating a dialogue between researchers. Additional GPT-4o conversations were later conducted to assess the coherence of prior outputs, verify citation accuracy, and evaluate the novelty of suggested research ideas in comparison to existing literature. The iterative nature of this process allowed for continuous refinement and ensured that the generated insights were relevant and valuable for further research.

The first GPT-4o conversation involved 25 questions aimed at gathering background information and summarizing key areas of research, focusing initially on zoonotic diseases and natural products. ChatGPT proposed additional relevant topics, some aligning with ongoing research while others introduced new directions such as pathogen dynamics. After refining these insights, ChatGPT produced a two-page summary, later expanded to four pages. In the second conversation, seven additional questions explored bioinformatics applications in marine and human health sciences, leading to five core topic areas, including marine animals as disease reservoirs and bioinformatics tools for environmental and human health. This process further identified emerging topics like regenerative medicine, and marine pollution’s impact on human health. The final output was structured as shown in the example below to outline each area’s research potential, needs, and impact, providing a foundation for future exploration:

#### Microbial Ecology and Environmental Resilience.

*Future Potential: Further exploration of microbial adaptations in marine environments, particularly in response to extreme or shifting conditions, could provide novel insights into resilience mechanisms. For instance, understanding how marine microbes recover from pollution events could inform approaches to enhance the resilience of the human microbiome during health disruptions, such as antibiotic treatments or chronic disease. Expanded comparative studies between marine and human microbiomes may identify shared pathways and microbial functions that could guide therapeutic interventions.*

*Research Needs: Development of more comprehensive bioinformatics tools to model microbial interactions and predict resilience under environmental stress. Integration of metagenomics and metabolomics to map functional contributions of marine microbes to ecosystem recovery. Potential Impact: Insights into microbial ecosystem resilience could lead to novel probiotics or bioremediation technologies.*

*References for Context.*

#### Expert commentary.

The intricate relationship between human health and the environment is increasingly evident as we face shared challenges in a rapidly changing world. Further GPT-4o conversations were conducted to evaluate whether the model could detect similarities to other sources and ensure originality, with no direct copying identified, though there was some difficulty in verifying a subset of references. After the release of ChatGPT-4.5, the initial analysis was repeated in both 4o and 4.5 omitting three redundant redundant questions and the output from five questions was compared. While the exact text in the responses was different, the content remained topically similar and useful for later human review to validate accuracy and cited references.

## 3. Discussion

This paper used several publicly available tools for the automatic creation of reviews. Each tool and method had its unique use cases, strengths, and some potential limitations, which we will discuss below.

In method 1, NotebookLM proved to be a valuable tool for conducting a narrative review of manually preselected articles. The platform effectively summarized information from 10 out of 12 provided sources, while omitting data from 2 citations demonstrating selectivity in its summarization effort, which is a limitation for scoping and systematic review. In addition, NotebookLM tried to incorporate in several instances information from the references section of reviewed citations by looking at the titles of references. This wasn’t expected as part of the original instruction (prompt) to LLM and can be seen as a false positive type of error. The intuitive interface of NotebookLM allowed for a seamless review process. However, the tool’s limitations include the need for manual selection of data sources, support for a maximum of 50 citations, and optimal performance may be limited to even smaller citation loads. These constraints highlight that while NotebookLM is efficient and easy to use, it requires careful curation and might not be suitable for larger-scale reviews.

In method 2, scite.ai was an effective tool for providing a review of the field of natural products that accurately described major contributions of the field to drug discovery. The strength of this tool is its ability to paint with broad strokes and package a wide subject matter into a concise report. However, it is limited in its capacity to provide a nuanced description of technical details or to qualify broad claims with caveats, unless explicitly requested in the prompt. Thus, careful construction of the prompt is very important for balanced results. The citations reviewed by scite.ai were representative of the field and comprised well-cited and impactful articles. Additionally, the citations were accurately integrated into the text. The drawback here was with input style of the prompts. Each prompt initiates a new inquiry into the literature which often results in redundancy between each where additional references would have strengthened the output. Generally, scite.ai was a constructive tool for quickly exploring ideas in a way that is supported by well cited and annotated research articles. There were no obvious hallucinations. In fact, the generated responses were coherent and easy to follow, although the writing style was rote at times and benefited from editing to qualify statements and adding segways. As a tool for generating detailed reviews, scite.ai still requires the input of a subject matter expert. However, as a tool for an expert to search the literature in a way that provides references that a traditional “keyword” search may miss, it is effective and can be used productively.

In method 3, the use of a conversational LLM model helped explore the current status of research outside that inputted through preselected published works. This offered unique advantages in developing comprehensive summaries of current and future research topic areas not dependent on specific citations. While there are an increasing number of LLMs available, ChatGPT was selected for this review due to its availability as part of a personal subscription and used with the goal of developing a list of potential research opportunities to bridge gaps between the marine environmental and human health sciences. ChatGPT provided helpful suggestions leveraging advances in technologies, computational tools, and integrative frameworks to address critical questions about microbial resilience, therapeutic potential, and pollutant impacts among others. These ideas were intuitive and the speed at which ideas could be suggested and challenged provided a valuable opportunity to identify new research priorities focusing on transformative discoveries that could benefit both marine ecosystems and human health. ChatGPT was especially helpful for developing these summaries and future research topics in terms of background, speed, and professionally formatted output.

## 4. Conclusion

The emerging area of automatic scientific review creation combined with expert validation appears to be a viable approach to scientific knowledge extraction. This approach uses the principles of the fundamental theorem of biomedical informatics [ 47 ] by empowering experts with computer systems to improve their abilities in their respective areas.

### Disclaimers

U.S. National Oceanic and Atmospheric Administration Disclaimer: The scientific results and conclusions, as well as any opinions expressed herein, are those of the author(s) and do not necessarily reflect the views of NOAA or the Department of Commerce. The mention of any commercial product is not meant as an endorsement by the Agency or Department.

### Appendices

- Appendix 1. ChatGPT – initial questions
- Appendix 2. ChatGPT – secondary questions
